# Orthopedic Surgical Treatment of Patients with Tumor‐induced Osteomalacia Located in the Hip Bones: A Retrospective Analysis of 10 Years in a Single Center

**DOI:** 10.1111/os.14105

**Published:** 2024-06-17

**Authors:** Shuzhong Liu, Xi Zhou, Annan Liang, Jinyi Xing, Yong Liu, Jin Jin, Jianguo Zhang, Weibo Xia

**Affiliations:** ^1^ Department of Orthopaedic Surgery Peking Union Medical College Hospital, Peking Union Medical College and Chinese Academy of Medical Sciences Beijing China; ^2^ Department of Endocrinology, National Health Commission Key Laboratory of Endocrinology, State Key Laboratory of Complex Severe and Rare Diseases Peking Union Medical College Hospital, Chinese Academy of Medical Sciences & Peking Union Medical College Beijing China

**Keywords:** Orthopedic surgery, Tumor‐induced osteomalacia, Treatment strategy, Hip bone

## Abstract

**Objective:**

The orthopedic surgical treatment strategies for patients with tumor‐induced osteomalacia (TIO) require improvement, especially for patients where the causative tumors are located in surgically challenging areas, requiring a greater degree of in‐depth investigation. This work aims to summarize and investigate clinical features and orthopedic surgical treatment effects of patients with tumor‐induced osteomalacia (TIO), whose causative tumors are located in the hip bones.

**Methods:**

A retrospective analysis was conducted on the clinical data of all patients diagnosed with culprit tumors located in the hip bones who underwent surgical treatment at the orthopedic bone and soft tissue tumor sub‐professional group of Peking Union Medical College Hospital from January 2013 to January 2023. This retrospective study summarized the clinical data, preoperative laboratory test results, imaging findings, surgery‐related data, perioperative changes in blood phosphorus levels, and postoperative follow‐up data of all patients who met the inclusion criteria. Normally distributed data are presented as mean and standard deviation, while non‐normally distributed data are shown as the means and 25th and 75th interquartile ranges.

**Results:**

The clinical diagnostic criteria for TIO were met by all 16 patients, as confirmed by pathology after surgery. Among the 16 patients, we obtained varying degrees of bone pain and limited mobility (16/16), often accompanied by difficulties in sitting up, walking, and fatigue. An estimated 62.5% (10/16) of patients had significantly shorter heights during the disease stages. All 16 patients underwent surgical treatment for tumors in the hip bones, totaling 21 surgeries. In the pathogenic tumor, there were 16 cases of skeletal involvement and none of pure soft tissue involvement. Out of the 16 patients, 13 cases had a gradual increase in blood phosphorus levels following the latest orthopedic surgery, which was followed up for 12 months to 10 years. Due to unresolved conditions after the original surgery, four patients received reoperation intervention. Two cases of refractory TIO did not improve in their disease course.

**Conclusion:**

In summary, the location of the causative tumor in the hip bone is hidden and diverse, and there is no defined orthopedic surgical intervention method for this case in clinical practice. For patients with TIO where the tumors are located in the hip bones, surgical treatment is difficult and the risk of postoperative recurrence is high. Careful identification of the tumor edge using precise preoperative positioning and qualitative diagnosis is crucial to ensure adequate boundaries for surgical resection to reduce the likelihood of disease recurrence and improve prognosis.

## Graphical abstract

- The location of the causative tumor in the hip bone is hidden and diverse, resulting in great challenges to endocrinologists and orthopedists.
- For the intraosseous tumor in hip bone, it may be quite difficult to remove the culprit tumor. In this situation, the proportion of recurrent/refractory cases has significantly increased, posing a huge challenge to the diagnosis and treatment of patients with this type of disease.

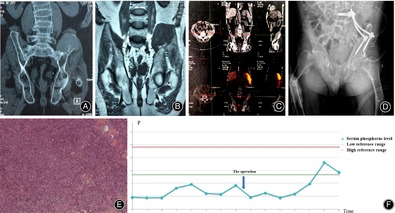

## Introduction

Tumor‐induced osteomalacia (TIO) is an uncommon paraneoplastic syndrome characterized by tumor‐induced increased renal phosphorus excretion resulting in acquired hypophosphatemic osteomalacia.^1^, ^2^, ^3^ The major clinical signs include muscle weakness, bone pain, and in severe cases, skeletal deformities, pathological fractures, and mobility disorders, which have a substantial impact on the quality of life and normal life among patients.^4^, ^5^ Over 1000 cases of TIO have been reported globally since McCance reported the first case in 1947.^3^, ^4^, ^5^, ^6^ The early disease stages can often be misdiagnosed as ankylosing spondylitis, primary osteoporosis, or spinal degeneration.^1^, ^2^ TIO‐causing tumors can be surgically removed with an accurate diagnosis and identification, typically resulting in an effective correction of hypophosphatemia and significant alleviation of symptoms.

Determination of the tumor location as well as the improvement of clinical symptoms and laboratory indicators following surgical resection are key to establishing TIO diagnosis.^7^, ^8^ Disease‐causing tumors are widely distributed in the human body and can arise in subcutaneous tissues, limb bones and joints, spine, soft tissues, palms, and toes. However, they are most prevalent in limb bones and soft tissues.^1^, ^2^, ^3^ More than half of intraosseous tumors that cause TIO are found in the long bones of the lower extremities, and a majority of them grow eccentrically. A few tumors involve the cortical bone, causing its osteolytic destruction. At present, various imaging techniques can be used to localize this type of tumor, including ultrasound, whole‐body MRI, octreotide SPECT–CT, and ^68^Ga‐DOTATATE positron emission tomography and computed tomography (PET/CT).^1^, ^2^, ^3^, ^9^ The causative tumors for TIO are mostly benign tumors originating from mesenchymal tissue, with lesions located within bone or soft tissue. For some TIO patients, the location of the culprit tumor is hidden and the culprit tumor may grow slowly, complicating diagnosis or delaying treatment. The orthopedic surgical strategy for patients with TIO is a recent development, and the diagnosis, treatment, and prognosis of patients with causative tumors in different locations may vary.^10^ This is especially true when tumors are situated in surgically difficult regions, such as the hip bones, and presents challenges for orthopedic surgeons. Therefore, the surgical treatment of such tumors is an important issue that requires further in‐depth research.

This study primarily focuses on clinical characteristics, early diagnosis, and treatment of TIO's causative tumor in the hip bone. The results of this study will: (i) provide an important reference for the development of orthopedic surgical expertise for the treatment of TIO patients with culprit tumors located in surgically challenging areas, such as the hip bones; (ii) improve the intraoperative procedures and the level of treatment for TIO patients with causative tumors located in the hip bone region, including determination of the tumor resection boundary; and (iii) further enhance diagnostic and therapeutic expertise to improve both the clinical diagnosis and treatment level of patients with culprit tumors located in surgically challenging areas such as the hip bones.

## Materials and Methods

### Inclusion and Exclusion Criteria

Inclusion criteria included: (i) clinical manifestations with continuously worsening bone pain symptoms, often starting at weight‐bearing joint sites (including feet, hips, knees, ankles, chest and waist); (ii) laboratory examination with preoperative existing hypophosphatemia; (iii) bone pain symptoms gradually improving and blood phosphorus levels rapidly increasing after resection of oncogenic tumors; (iv) imaging examination through magnetic resonance imaging or CT, whole body bone imaging, octreoscan or octreotide SPECT–CT, or ^68^Ga‐DOTATATE PET/CT examination of the responsible lesion, the location and adjacent relationship of the culprit tumor can be detected, confirming that the suspected pathogenic tumor is located in the hip bone and surrounding area; and (v) Pathological examination confirming that the tumor responsible for resection is pathogenic tumor of TIO.

Exclusion criteria included: (i) patients who have not received surgical treatment or have surgical contraindications; (ii) cases with incomplete clinical data; and (iii) osteomalacia caused by other factors.

### General Information

This study included surgical treatment‐related data from all consecutive patients with TIO located at the hip bone region who underwent surgical treatment in the orthopedic bone and soft tissue tumor sub‐professional group of Peking Union Medical College Hospital from January 2013 to January 2023. The age, gender, symptoms and signs, bone pain location, height shortening during the disease stage, the occurrence of pathological fractures during the course, and history of orthopedic surgery unrelated to causative tumors after disease onset were statistically analyzed. We also analyzed information on the distribution of pathogenic tumor sites.

#### Laboratory Examination, Imaging Examination, and Pathological Diagnosis

##### Laboratory Examination

We analyzed blood phosphorus, blood calcium, alkaline phosphatase (ALP), urine phosphorus, urine calcium, 24‐h urine phosphorus, 24‐h urine calcium, parathyroid hormone (PTH), blood 25‐(OH)D, blood 1,25‐(OH)_2_‐D, and the carboxy‐terminal cross‐linking telopeptide of type I collagen (beta‐CTX) of all patients.

##### Imaging Examination

Preoperative bone density, limb and trunk X‐ray, surgical area B‐ultrasound, CT, MRI, whole‐body bone imaging, octreoscan or octreotide SPECT–CT, ^68^Ga‐DOTATATE PET/CT examination for qualitative and localized diagnosis, assisting in the formulation of surgical plans and the assessment of surgical risks.

##### Pathological Diagnosis

The excised tumor tissue samples were sent for examination during surgery. After surgery, the tumor tissue sections were removed for H.E. staining and immunohistochemistry staining. The nature and pathological characteristics of the lesion were diagnosed by an experienced physician from the Department of Pathology.

The normalization of blood phosphorus levels in patients after surgery is considered indicative of effective surgical resection. In cases where disease recurrence or unresolved symptoms are observed during the follow‐up process, further localization or other treatment options should be performed according to the diagnosis and treatment flowchart of the TIO orthopedic surgical treatment strategy.^10^

#### Statistical Analysis

All normally distributed data of this study were described with mean and standard deviation. Non‐normally distributed data were described as the median and 25th and 75th interquartile ranges (Q25, Q75). A MedCalc Statistical Software version 15.2.2 (MedCalc Software, Ostend, Belgium) was used to complete data processing and statistical analyses.

## Results

### General Characteristics

**FIGURE 1:**
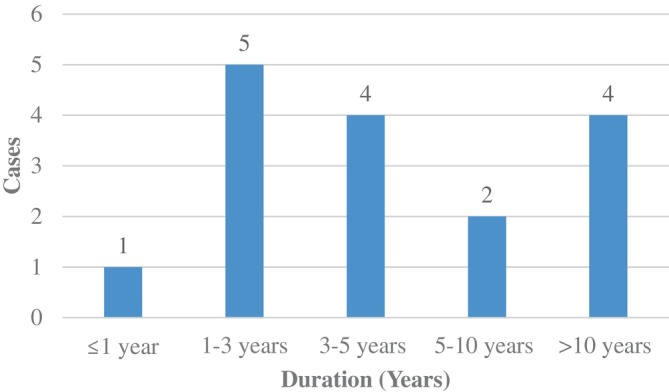
Time distribution map of disease progression from onset to diagnosis for all patients included in this study.

**FIGURE 2:**
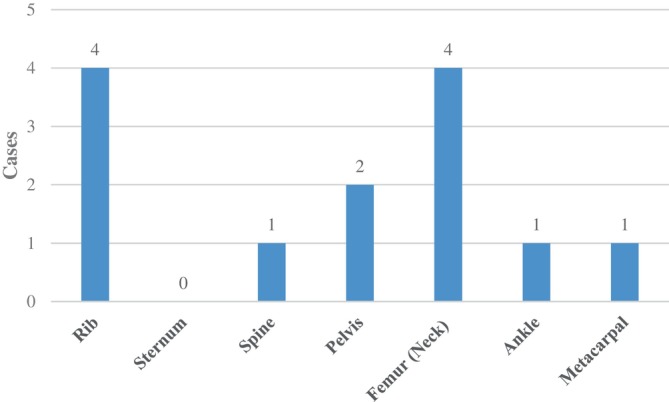
The distribution diagram of the locations where fractures/insufficiency fractures occurred in patients included in this study during the course of the disease.

Table 1 presents the basic clinical characteristics of all 16 patients. In total, nine male and seven female patients with a mean age (and standard deviation) of 41.5 ± 11.0 years (range, 24 to 55 years) were included in this retrospective study. The mean duration of symptoms before initial accurate diagnosis was 80.8 (30.0, 126.0) months (range, one to 25 years) (Figure 1). The follow‐up duration was from 12 to 120 months. Clinical symptoms included bone pain (100%), fractures (56.3%) (Figure 2), and muscle weakness or fatigue (93.8%).

**TABLE 1:** General data and clinical manifestations of TIO patients with culprit tumors located in the hip bones.

Cases	Age (y)	Sex	Symptoms	Disease course (m)	Bone pain sites	Difficulty in sitting up and walking	Fatigue	Limited mobility	Short stature	Pathological fractures	History of orthopedic surgery
1	47	M	Multiple bone pain occurred in the whole body for more than 8 years, and blood phosphorus decreased for more than 5 years	96	Bilateral thoracic ribs and lower fibula of left lower limb	Yes	Yes	Yes	Yes	None	None
2	35	F	Bone pain for more than 4 years	44	Left ankle and back of foot, left hip, ribs, right hip	Yes	Yes	Yes	Yes	Yes	None
			Bone pain was significantly relieved. From 2016 to April, walking with crutches can be ordered. After standing for about 2 h, knee, hip and waist pain occurred	56	Knees, hips, waist	Yes	Yes	Yes	Yes	Yes	None
3	33	M	Bone pain for more than 4 years	56	Left heel, waist and lower thoracic vertebrae	Yes	Yes	Yes	—	None	None
4	49	M	Bone pain and fatigue for 14 years	168	Back, limb joints, bilateral hip joints, lower limbs, thorax	Yes	Yes	Yes	Yes	Yes	None
	50	M	Intermittent back pain, self‐standing after squatting and hard to get up after lying down	180	Back	Yes	Yes	Yes	Yes	Yes	None
	53	M	Fatigue at night and pain in the lumbar spine when the range of motion is large	204	Waist	Yes	Yes	Yes	Yes	Yes	None
5	52	M	Multiple bone pain in the whole body for 3 years	36	Left foot	Yes	Yes	Yes	None	Yes	None
6	39	M	Bilateral iliac pain for more than 4 years, aggravated with multiple bone pain for more than 2 years	39	Bilateral ilium, lumbar vertebrae, thoracic vertebrae, thorax, ribs, spine, thorax, lower limbs and left ankle joint	Yes	Yes	Yes	—	None	None
7	55	M	More than 25 years of systemic multiple bone pain	300	Ribs, bilateral acetabulum, long bones of limbs and spine, bilateral knee joints	Yes	—	Yes	—	Yes	Yes
8	54	F	Bone pain, short stature for 13 years, progressive exacerbation for 2 years	156	Heels, lower limbs and waist and back	Yes	Yes	Yes	Yes	Yes	None
9	41	F	Progressive pain of both lower limbs for more than 2 years and fatigue for more than 1 year	24	Pain in the dorsum of both feet, lateral side of right leg, groin of right lower limb, lateral side of left knee joint and left shoulder joint	Yes	Yes	Yes	—	Yes	None
10	57	F	General bone pain for 3 years	36	Right quarter rib, heels, lower limbs, back and left hip	Yes	Yes	Yes	Yes	None	None
11	49	M	Progressive aggravation of bone pain occurred for 2 years and cystic space occupying lesion of right iliac joint was found for 5 months	24	Lateral right foot, ankle joint, knee joint, hip joint, waist and back, anterior chest ribs, shoulders, metacarpophalangeal joint of right thumb, interphalangeal joint of left thumb	Yes	Yes	Yes	Yes	Yes	None
12	28	F	General weakness for 7 years, bone pain for 4 years	84	Waist, limbs, chest and rib	Yes	Yes	Yes	Yes	None	None
						Yes	Yes	Yes	Yes	None	None
13	24	F	Multiple bone pain and fatigue in the whole body for 1 year	12	Bilateral hip joint and its surroundings, bilateral knee joint, bilateral costal region	Yes	Yes	Yes	Yes	None	None
	24	F	Fatigue, bone pain, height loss for 2 and a half years	30	Bilateral ribs	Yes	Yes	Yes	Yes	None	None
14	26	F	Bone pain for more than 3 years	38	Left posterior costal region, bilateral posterior costal region, bilateral lumbosacral region, dorsum of both feet, right hip joint	Yes	Yes	Yes	Yes	None	None
15	45	M	Bone pain and fatigue for more than 2 years, aggravated in the past 7 months	24	Lumbar back, double lower ribs, double dorsalis pedis, double hips, double shoulder joints	Yes	Yes	Yes	None	Yes	None
16	30	M	General bone pain with fatigue for 13 years	156	Lumbosacral, shoulder, elbow, wrist, right thigh	Yes	Yes	Yes	Yes	Yes	Yes

*Note*: “—” represents the information is not available.

Abbreviations: F, Female; M, Male; m, month.

### Qualitative and Localized Diagnosis

#### Laboratory Examination

All patients had varying degrees of hypophosphatemia before surgery (Table 2) Preoperatively, the average blood phosphorus level of patients included in this study was 0.41 ± 0.10 mmol/L.

**TABLE 2:** Tumor characteristics, surgical intervention, and postoperative recovery of all patients involved in this study.

Cases	Age (y)	Sex	Preoperative blood phosphorus(mmol/L)	Localization	Tumor size (cm)	Operation	Outcome	Complication	Histological diagnosis	Postoperative hospitalization time (d)
1	47	M	0.48	Proximal posterior margin of left iliac bone	2.0 × 2.0 × 1.0	Left acetabular tumor resection and bone cement reconstruction surgery	Remission	Drop foot	PMT	8
2	35	F	0.35	The junction of the anterior wall of the right acetabulum and the pubic ramus	1.0	Right acetabular tumor resection and bone cement reconstruction surgery	No remission	None	PMT	17
			0.41	Right hip area medial to pubic ramus	2.0	Right acetabular mass enlargement resection and bone cement reconstruction through the ilioinguinal approach	No remission	None		3
3	33	M	0.35	Posterior margin of right acetabulum	2.0 × 2.0 × 1.0	Right acetabular tumor resection and bone cement reconstruction surgery	Remission	None	PMT	5
4	49	M	0.35	Posterior margin of left acetabulum	1.0 × 1.0 × 1.0	Left acetabular lesion resection and bone cement reconstruction through posterior approach	No remission	Left foot dorsiflexion limitation	PMT	6
	50	M	0.62	Posterior part of left acetabulum (lateral edge above the original operation area)	1.0 × 1.0 × 1.0	Left acetabular lesion resection and bone cement reconstruction through posterior approach	No remission	None	PMT	5
	53	M	0.52	Left iliac bone (at the upper edge after bone cement)	1.0 × 10 × 1.0	Left acetabular lesion resection and bone cement reconstruction through posterior approach	No remission	None	PMT	6
5	52	M	0.42	Left iliac bone	1.0 × 1.0	Left pelvic lesion clearance+bone cement reconstruction surgery	Remission	None	PMT	6
6	39	M	0.64	Right acetabulum	3.0 × 2.0 × 1.0	Front approach right acetabular lesion curettage and bone cement reconstruction surgery	Remission	None	PMT	6
7	55	M	0.36	Right acetabulum	1.0 × 1.0 × 1.0	Right acetabular lesion resection and bone cement reconstruction through posterior approach	Remission	None	PMT	4
8	54	F	0.34	Upper left acetabulum	1.0 × 1.0 × 0.5	Front approach left acetabular lesion scraping and bone cement reconstruction surgery	Remission	None	PMT	6
9	41	F	0.33	Right acetabulum	1.0 × 1.0 × 1.0	Right acetabular lesion resection and bone cement reconstruction through posterior approach	Remission	None	PMT	8
10	57	F	0.48	Left acetabulum	—	Percutaneous puncture biopsy of left acetabular lesion, bone cement filling and sterilization surgery	Recurrence	None	PMT	6
11	49	M	0.45	Right acetabulum	2.0 × 2.0 × 1.0	Excision of lesion around the right acetabulum and reconstruction with bone cement	Remission	Right hip infection	PMT	8
12	28	F	0.63	Left iliac bone (left iliac bone near sacroiliac joint)	—	Percutaneous left iliac bone lesion puncture biopsy and bone cement filling surgery	No remission	None	PMT	23
				Left iliac bone (left iliac bone near sacroiliac joint)	—	Left iliac bone lesion resection, internal fixation, and bone grafting reconstruction surgery	Remission	None	PMT	9
13	24	F	0.23	Left acetabulum	2.1 × 1.8	Posterior approach left acetabular lesion resection and bone cement reconstruction surgery	No remission	None	PMT	15
	24	F	0.26	Left acetabulum	4.1 × 3.1	Hip joint lesion resection surgery + bone cement	Remission	None	PMT	5
14	26	F	0.40	Left acetabulum		Posterior approach left acetabular lesion resection and bone cement reconstruction surgery	Remission	None	PMT	5
15	45	M	0.40	Left iliac bone	1.0 × 1.0 × 1.0	Partial iliac resection surgery and left iliac bone tumor resection, bone cement reconstruction surgery	Remission	None	PMT	3
16	30	M	0.36	Middle part of left iliac wing	6.0 × 5.0 × 4.0	complete pelvic tumor resection (type I, Enneking classification) with internal fixation and reconstruction	Remission	None	PMT	14

*Note*: “—” represents the information is not available.

Abbreviations: cm, centimeter; d, day; F, Female; M, Male; PMT, phosphaturic mesenchymal tumor.

#### Localization of the Causative Tumor

**FIGURE 3:**
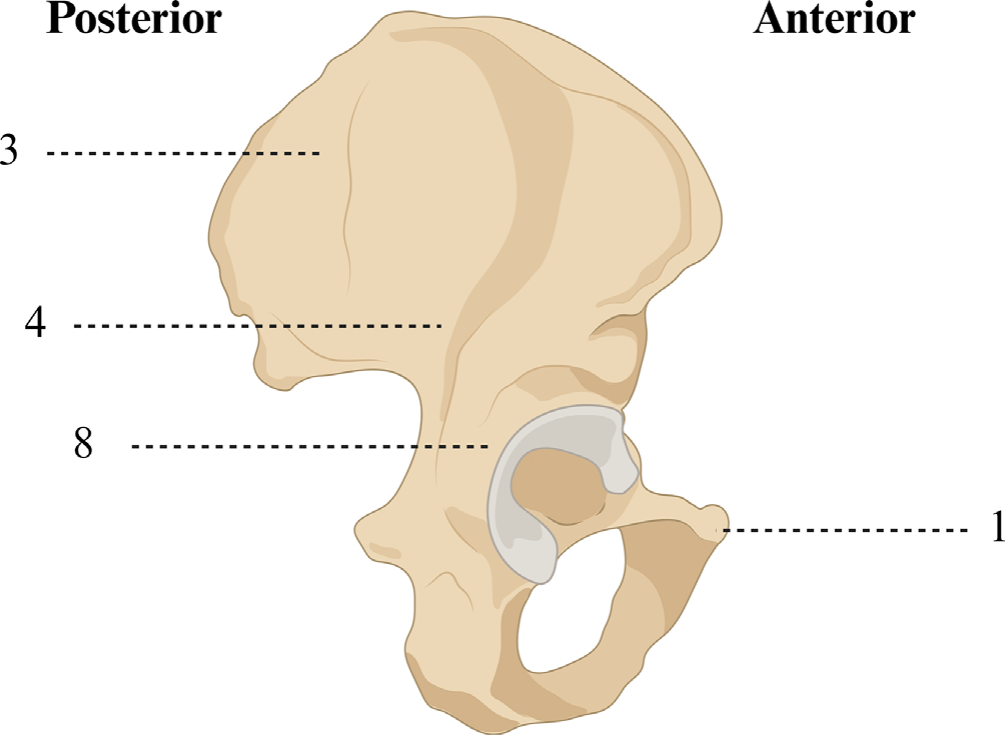
Anatomical distribution map of the hip bones where the responsible tumors were located (Created with BioRender.com, and the license to publish from the BioRender has been obtained).

All causative tumors were identified by a stepwise approach involving multiple imaging modalities. Before surgery, 14 patients underwent octreoscan or octreotide SPECT–CT, and 13 patients completed PET/CT (11 patients for ^68^Ga‐DOTATATE PET/CT). Additionally, 14 patients completed the MRI examination of the location of the pathogenic tumor, and the other two patients underwent a CT examination of the lesion site. The anatomical schematic diagram shown in Figure 3 shows the distribution of culprit tumors in all 16 patients with TIO.

#### Surgical Treatment

As shown in Table 2, 16 patients underwent 21 surgeries, with four patients undergoing second or third operations for unresolved or relapsed conditions. One female patient (patient two) accepted parathyroidectomy due to tertiary hyperparathyroidism. Thereafter, her serum calcium normalized, whereas, serum P remained low. The overall cure rate was 81.3% (13/16) for surgical treatment of TIO with causative tumors in the hip bones.

#### Pathological Diagnosis and Postoperative Follow‐up

The histopathology results of tissues from all 21 surgeries (100%) were consistent with phosphaturic mesenchymal tumors. The histologic features of a phosphaturic mesenchymal tumor included tumor cells infiltrating between bone trabeculae; bland, spindled to stellate‐shaped cells with extremely few mitoses in most cases; a very well‐developed capillary network; and calcification of the matrix of the tumor in an unusual “grungy” or flocculent fashion.

**FIGURE 4:**
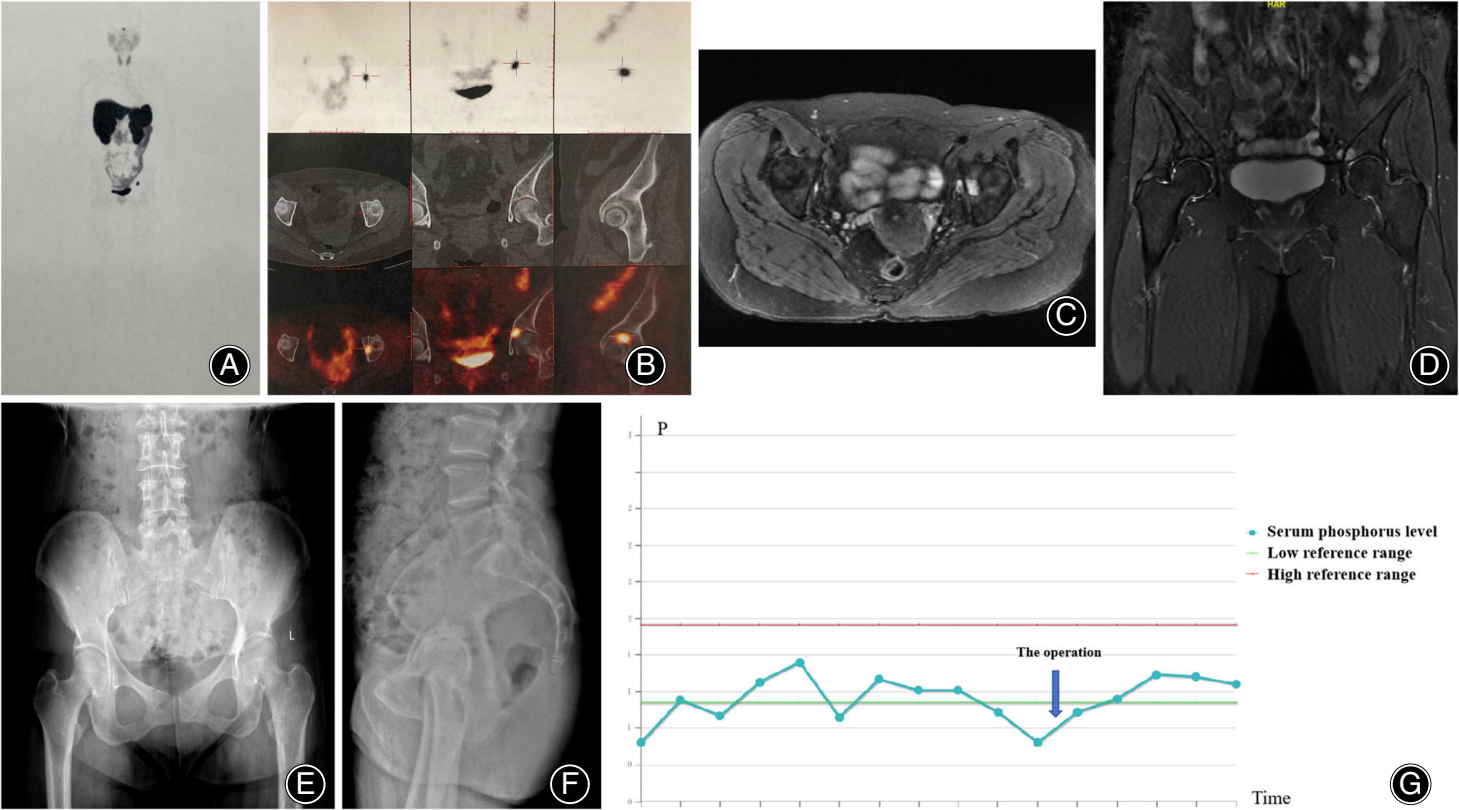
Case 1 (A) The octreoscan showing the suspected culprit tumor in left acetabular region. (B) The ^68^Ga‐DOTATATE PET/CT revealing high intake in the left acetabulum. (C, D) MRI of the hip revealing the mass, which was highly indicative of the causative tumor. (E, F) The postoperative X‐ray of the pelvis. (G) Serum phosphorus levels elevated to the normal range after the operation.

**FIGURE 5:**
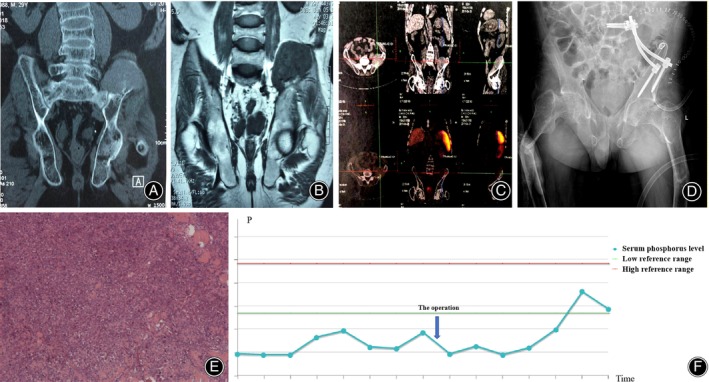
Case 2 (A) CT of the hip showing the suspected culprit tumor of the left acetabulum. (B) MRI of the hip revealing the suspected causative tumor. (C) The first‐line ^68^Ga‐DOTATATE PET/CT scan showing high intake in the left acetabulum. (D) Postoperative X‐ray demonstrating the position of the internal fixation was satisfactory. (E) Pathological results confirmed the diagnosis of phosphaturic mesenchymal tumor. (F) Serum phosphorus levels elevated to the normal range postoperative.

In most cases, the serum P level normalized after a mean of 5.9 ± 2.7 days (range, 2–11 days) postoperatively (Table 3). The follow‐up period ranged from 12 months to 10 years. Among the 16 patients, only two patients experienced limited left foot dorsiflexion after surgery and one patient suffered from right hip infection after the operation. Additionally, all 16 patients included one recurrent case and two refractory TIO cases. Among the 16 patients, 15 underwent lesion resection and bone cement reconstruction (Figure 4), whereas one patient underwent complete pelvic tumor resection (type I, Enneking classification) and was subsequently clinically cured (Figure 5). For cases which postoperative recovery does not improve, neutral phosphorus solution and active vitamin D treatment was administered, and the condition was closely monitored. So far, patients with TIO, managed by our team were regularly followed up in the orthopedic clinic and endocrinology, and their condition is relatively stable.

**TABLE 3:** Changes of blood phosphorus level in TIO patients with culprit tumors in the hip bones.

Cases	Age (y)	Sex	Preoperative P(mmol/L)	Postoperative day 1 (mmol/L)	Postoperative day 2 (mmol/L)	Postoperative day 3 (mmol/L)	Postoperative day 4 (mmol/L)	Postoperative day 5 (mmol/L)	Postoperative day 6 (mmol/L)	Postoperative day 7 (mmol/L)	Postoperative day 8 (mmol/L)	Postoperative day 9 (mmol/L)	Postoperative day 11 (mmol/L)
1	47	M	0.48	0.52	0.64	0.44	0.38	0.47	0.56	0.68	0.81		
2	35	F	0.35	0.36		0.34		0.37	0.41	0.44		0.4	
			0.41	0.34	0.27	0.33			0.36		0.36		
3	33	M	0.35	0.47		0.81	0.93						
4	49	M	0.35	0.27	0.27	0.26	0.33	0.43	0.44				
	50	M	0.62	0.34	0.4	0.39	0.43	0.47					
	53	M	0.52		0.46	0.56							
5	52	M	0.42	0.52	0.46	0.49		0.88					
6	39	M	0.64	0.74	0.72			0.89					
7	55	M	0.36	0.38	0.46	0.57							
8	54	F	0.34	0.41	0.53	0.72	0.86		0.93				
9	41	F	0.33	0.44	0.48	0.49	0.52		0.73	0.78	0.93		
10	57	F	0.48	0.73	0.84	1.04	1.02		0.96				
11	49	M	0.45	0.51	0.55	0.77			1.09	0.97			
12	28	F	0.63	0.5	0.52	0.44			0.53				
				0.56				0.94			1.11		
13	24	F	0.23	0.21	0.19	0.23			0.26	0.32	0.33		
	24	F	0.26	0.24		0.56			0.84				
14	26	F	0.40	0.32		0.6	0.73						
15	45	M	0.4	0.57	0.65								
16	30	M	0.36	0.55	0.27	0.37		0.26		0.35		0.59	1.08

Abbreviations: M, Male; F, Female.

## Discussion

For patients with tumor‐induced osteomalacia, symptoms can often be significantly alleviated after surgical resection of oncogenic tumors.^11^, ^12^, ^13^ To date, most research has focused on the disease pathogenesis, changes in bone metabolism, preoperative diagnosis, and postoperative follow‐up of patients with TIO. Relatively little attention has been paid to orthopedic surgical treatment strategy, despite its importance in patient prognosis. In April 2024, our team authored the first review of the preoperative evaluation and orthopedic surgical strategies used for patients with TIO, published in the *Journal of Bone Oncology*.^10^ In this study, we summarized and analyzed the clinical features and orthopedic surgical treatment effects of patients with TIO in our institution, whose culprit tumors are located in the hip bones.

### Preoperative Evaluation of Patients with TIO Where the Culprit Tumor is Located in the Hip Bones

Hypophosphatemia can often be efficiently treated, and symptoms significantly alleviate after complete surgical removal of TIO's causative tumors after precise characterization and localization.^11^, ^12^, ^13^ Most of the causative tumors are benign, and a majority can be cured after complete surgical resection. Therefore, it is particularly important to actively search for the responsible tumor. The majority of TIO was found in the limbs, followed by the head, neck, and maxillofacial region. Patients with culprit tumors located in the hip bone region are relatively rare, and the local anatomical structure is complex, which poses certain difficulties in qualitative localization diagnosis. A comprehensive physical examination combined with several auxiliary examinations, including ultrasound, CT, MRI, octreotide SPECT–CT, ^68^Ga‐DOTATATE PET/CT is required for the localization of primary tumors.^14^, ^15^, ^16^ Because somatostatin receptor is highly expressed in most of these tumors, octreotide imaging (somatostatin receptor tracer imaging) is fundamental for the localization of preliminary tumors. However, octreotide SPECT/CT and ^68^Ga‐DOTATATE PET/CT examination are inadequate for locating lesions; it cannot fully meet the needs of a preoperative understanding of the relationship between the tumor and its surrounding tissues. Therefore, further information on the tumor can be obtained through ultrasound, MRI or CT examination to precisely guide operation.^14^, ^15^, ^16^ Of note, not all tumors have somatostatin receptor expression on the surface; moreover, both the literature and our experience confirmed that the negative octreotide scan and ^68^Ga‐DOTATATE PET/CT examination could not completely exclude the possibility of TIO. Surgical treatment is the first choice after accurately locating a suspicious causative tumor.^1^, ^2^, ^3^, ^11^, ^12^ Before identifying the primary tumor, oral neutral phosphorus preparation and oral active vitamin D are temporarily necessary to improve clinical symptoms. Blood phosphorus may increase to a normal range in this case.

### Surgical Tips for Patients with TIOs with Culprit Tumors Located in Hip Bones

Hip bone has a complex anatomical structure. The causative tumor for TIO in the hip bone is highly unusual in the clinic, with no previous international diagnosis and treatment reports as well as experience. Thus, clinical treatment is considerably challenging. In our investigation, the causative tumor of all patients had skeletal involvement. Based on our diagnosis and treatment experience, the postoperative recurrence rate and proportion of refractory TIO cases with bone involvement are significantly higher than those with soft tissue tumors.^11^, ^17^ Therefore, four patients in this study underwent reoperation and two refractory cases together with one recurrent case did not receive clinical cure in recent follow‐up. Table 4 shows a literature summary of previously reported cases of TIO with causative tumors located in the acetabular region.^18^, ^19^, ^20^, ^21^ At present, the clinical treatment principle of culprit tumor in hip bone is to eliminate the tumor and adjacent tumor tissue, reduce the risk of local recurrence, and incidence of complications, as well as maximize the recovery of limb function. However, due to the unique anatomical location of the tumor in hip bone, surgeons may need to perform hemipelectomy and other potentially damaging surgical procedures to completely remove the causative tumor.^18^, ^19^, ^20^, ^21^, ^22^ However, clinical experience suggests that even if the patients with TIO in the hip bones agree to open surgical resection, there is still a risk of local recurrence or negative pathological results. Therefore, selecting an acceptable surgical procedure for treating causative tumors in the hip bones and improving the effect of surgical treatment is a thorny problem confronted by orthopedics doctors.

**TABLE 4:** Clinical review of four previously published causative tumors for TIO in the acetabular regions.

Authors	Year	Age (y), sex	Symptoms	Laboratory findings	Localization methods	Localization	Tumor size	Treatment	Outcome	Follow up (m)	Histological diagnosis
Turin CG^18^	2021	43, M	Progressive fatigue, weakness, muscle and joint pain in setting of recurrent fractures	Normal calcium at 9.3 mg/dL, low phosphorus at 1.5 mg/dL, low 1,25‐dihydroxyvitamin D at 13 pg/mL, high phosphate excretion fraction in urine (27%), elevated ALP at 163 U/L, and elevated FGF23 at 238 RU/mL	^68^Ga‐DOTATATE PET/CT	The left acetabular	1.4 cm	Biopsy; radical resection of the tumor of the posterior left acetabulum with reconstruction of the posterior wall and posterior column of the acetabulum through a Kocher‐Langenbach approach	Complete resolution	6	Spindle cell neoplasm；phosphaturic mesenchymal tumor
Mishra SK^19^	2019	43, M	5‐year history of diffuse bony pains, pain in ribs, and proximal muscle weakness	Hypophosphatemia, elevated serum FGF‐23, low 1,25 (OH)_2_D, and decreased percentage of tubular reabsorption of phosphate.	^68^Ga‐DOTATATE PET/CT	The roof of right acetabulum	About 1.1 × 0.9 × 1.4 cm	A single session of radiofrequency ablation; A biopsy of the lesion was performed before ablation	Complete resolution	24	Scanty tissue
Nakamura K^20^	2018	68, F	Progressive bone pain of the rib cage, and polyarthralgia and back pain for 3 years	Hypophosphatemia (1.7 mg/dL) and an abnormally low level of 1,25(OH)_2_ vitamin D (15 pg/mL), hypophosphatemia (1.7 mg/dL), hypocalcemia (8.3 mg/dL), high serum ALP (1639 UI/L), high intact PTH (103.3 pg/mL), and low tubular resorption of phosphorus clearance (%TRP) (67.7%). The 1,25(OH)_2_ vitamin D3 level was 96.3 pg/mL. Importantly, the serum FGF‐23 level was elevated to 1500 pg/dL.	Bone scintigraphy, MRI, CT	The right acetabulum	—	An open biopsy of the bone tumor; Curettage of the tumor was performed and the defects were filled with bone allografts; Thereafter, total hip arthroplasty was performed. For the acetabular component, a Müller acetabular support ring was used since the bony defect of the acetabulum was large. A press‐fit type cementless femoral component was used	No remission; The patient started taking phosphate supplementation and alphacalcidol. Although a CT scan did not show findings of recurrence, the patient is being kept under careful observation.	12	PMT; loose proliferation of spin dle‐shaped cells without atypia accompanied by pericytoma tous vessels and microcystic change
Morimoto T^21^	2014	35, F	Lower back pain	High ALP levels (1080 mg/ml), hypophosphatemia (1.4 mg/ml), high thyroglobulin levels (65.2 mg/ml), normal calcium levels (8.7 mg/dl), normal PTH levels (58.7 mg/ml)	^18^F‐fluorodeoxyglucose	In the right acetabulum	—	An open biopsy; transcatheter arterial embolization (TAE) of the feeding artery of the pelvic tumor	The serum phosphate and ALP levels were gradually normalized. However, regrowth of the pelvic tumor and multiple metastases in the lung and bones were observed after the second TAE.	32	Malignant PMT

*Note*: “—” represents the information is not available.

Abbreviations: ALP, alkaline phosphatase; CT, computed tomography; F, Female; M, Male; m, month; MRI, magnetic resonance imaging; PET/CT, Positron emission tomography/computed tomography; PMT, phosphaturic mesenchymal tumor; PTH, parathyroid hormone.

Regarding surgical treatment, orthopedists should first establish the range, the implicated organs, and adjacent structures of the hip or acetabular tumor for TIO.^23^, ^24^, ^25^, ^26^ In cases where complete removal of the causative tumor is feasible, surgical boundaries are set at least 0.5 cm beyond the tumor margin. Surgeons must exercise discretion during the procedure, as overly conservative resection may lead to disease recurrence, while overly extensive resection may damage bone structure, affecting local stability and postoperative motor function. Furthermore, high‐temperature cautery around the lesion is an option to deactivate any residual tumors and minimize disease recurrence. For patients with phosphaturic mesenchymal tumor (PMT) in the hip bone, due to the complex anatomical structure of this region and the existence of local anatomical variations in patients, special attention should be paid to identifying important anatomical structures, including nerves, blood vessels, muscles, tendons, ligaments, and spermatic cords to prevent the occurrence of secondary injuries. In this study, two patients experienced limited foot dorsiflexion after surgery. The posterior or posterolateral approach is recommended due to the particularity of anatomical location. The full grasp of the local anatomical structure determine the incidence of intraoperative complications.

### Reconstruction Methods for Postoperative Bone Defect

Furthermore, methods of filling the residual bone defect following pelvic tumor resection or curettage, and reconstruction of the structure and function of the pelvis have presented challenges to orthopedic surgeons. For smaller local bone defects, bone cement filling and bone grafting may be more effective, while for large bone defects, the use of internal fixation or 3D‐printed prostheses is preferable for the restoration of biomechanical stability. However, further research is required to determine the optimal methods of bone defect repair after tumor resection in patients with TIO. For patients with surgical contraindications, those who do not accept open surgery to remove the causative tumor, or whose preoperative diagnosis is unclear, our team has conducted clinical research on the treatment of PMT in surgical‐challenging sites with bone cement tumor destruction technology in the past decade. The use of bone cement tumor destruction technology in the surgical treatment of patients with PMTs in surgical‐challenging cases may have the following benefits:^13^, ^27^ (i) bone cement tumor destruction technology can be completed under local anesthesia to reduce the risk of surgery; (ii) the application scope of bone cement tumor destruction technology can be further expanded, which can be used in reinforcing hip or acetabular structure; (iii) for patients diagnosed with PMT in hip bone, additional histopathological confirmation can be obtained; and (iv) the efficacy of this technology in the treatment of oncogenic tumors with bone involvement in certain specific areas has been confirmed. Therefore, minimally invasive cement destruction technology provides a novel idea and choice for early diagnosis and reasonable treatment of patients with PMTs in the hip bones.

### Postoperative Follow‐up

Postoperatively, patients should be closely followed up to monitor blood phosphorus levels. TIO patients should be regularly followed up and retested for the blood phosphorus, bone metabolism indexes, as well as postoperative symptoms.^8^, ^28^, ^29^, ^30^ Based on the experience of the guidelines, it is important to locate the suspected causative tumor for further diagnosis if the blood phosphorus levels remain lower than the normal value.^3^, ^4^, ^9^, ^17^ The standardized regular follow‐up after surgery is important, and whether the postoperative disease relapses or not, warrants the attention of orthopedic surgeons. For patients with tumor‐induced osteomalacia (TIO), surgical resection of the responsible tumor is the preferred treatment. In cases where surgical removal of the tumor is challenging or not feasible, image‐guided ablation offers a viable alternative, being effective, minimally invasive, and safe.^19^, ^31^ Cowan *et al*.^32^ suggested that CT‐guided cryoablation can expand the treatment options for TIO patients, particularly beneficial for those unwilling or unsuitable for surgery. Global guidelines for TIO recommend oral phosphate combined with active vitamin D or burosumab for cases where tumor resection is not possible, aiming to regulate calcium and phosphorus homeostasis, alleviate hypophosphatemia, and halt disease progression.^9^, ^33^ Cinacalcet may be considered for patients unable to tolerate phosphate solution or suffering from secondary hyperparathyroidism. Notably, recent research focus in TIO treatment has revolved around anti‐FGF‐23 monoclonal antibodies, such as burosumab, which bind to FGF23, inhibiting its activity and signaling pathways, thereby enhancing phosphorus reabsorption and serum vitamin D levels, ultimately improving bone mineralization.^34^, ^35^

### Strengths and Limitations

Despite presenting the extremely rare cases of PMT in the hip bone as well as summarizing the experience of diagnosis and treatment, this study still has many shortcomings: (i) first, the number of cases recruited in the analysis is relatively small, despite being the largest clinical analysis report of PMTs resulting from TIO location in the hip bone so far; (ii) this study is a single center study, though we have presented all the cases from our team over the past 10 years; (iii) this study is a retrospective study, which can lead to bias in the research results; and (iv) we have only reported therapeutic effect of short‐term and medium‐term follow‐up; the long‐term therapeutic effect warrants further confirmation.

## Conclusion

In conclusion, TIO with an oncogenic tumor in the hip bone is an uncommon bone metabolic disease, and its onset and progression will severely impact the quality of life of patients. The occurrence and progression of this disease will impose a significant physical and mental burden to patients, and a huge economic burden to society. Clinicians should improve their understanding of the disease, combined with clinical manifestations, imaging examination, pathological diagnosis, and other techniques to improve diagnosis accuracy and take reasonable therapy to improve prognosis. The etiology, pathogenesis, diagnosis, treatment, and prognosis of TIO need additional investigation to bring benefit to these patients.

## Conflict of Interest Statement

The authors declare that they have no competing interests.

## Ethics Statement

Written informed consent was obtained from the patient for publication of this article. Institutional ethical review and approval in Peking Union Medical College Hospital has been obtained with approval number S‐K1658.

## Author Contributions

All authors have full access to the data in this study, and take responsibility for the integrity of the data and the accuracy of the data analysis. Study concept and design: S.Z.L., X.Z., A.N.L., J.Y.X., Y.L., J.J., W.B.X. and J.G.Z. Manuscript preparation: S.Z.L., X.Z. Data collection and analysis: S.Z.L., X.Z., A.N.L., J.Y.X. Operation: Y.L. The patients' follow‐up: Y.L., J.J., and W.B.X. Manuscript revision for important intellectual content and technical details: Y.L., W.B.X. and J.G.Z. Obtained funding: S.Z.L.

## Data Availability

The anonymized data used and/or analyzed during the current study are available from the corresponding author on reasonable request.
